# What information do cancer patients want and how well are their needs being met?

**DOI:** 10.3332/ecancer.2018.873

**Published:** 2018-09-25

**Authors:** Gek Phin Chua, Hiang Khoon Tan, Mihir Gandhi

**Affiliations:** 1 National Cancer Centre Singapore, 11 Hospital Drive, 169610, Singapore; 2 Singapore Clinical Research Institute, 31 Biopolis Way Nanos, #02-01, 138669, Singapore

**Keywords:** patient information needs, cancer, communication, satisfaction, importance of information, sexuality, supportive care background

## Abstract

The goal of this study is to determine the type of information cancer patients need and to measure the extent to which these information needs are met by measuring patients’ levels of satisfaction. A self-administered questionnaire developed through extensive literature reviews was pilot tested on 11 cancer patients using convenience sampling in a large ambulatory cancer centre in Singapore. All eligible patients attending the centre during a 5-month period were invited to complete the 76-item survey that had been designed to evaluate self-reported information needs and level of satisfaction with the information received while undergoing cancer treatment. The importance of information and the level of satisfaction with needs being met were assessed with the 5-point Likert scale. A total of 411 patients (50%) completed the survey. Almost all patients wanted information about the disease, tests and investigations, treatment, side-effects, sexuality, psychosocial support and financial matters, and most items listed in the questions in each selection were rated as important or very important. Responses indicate that patients were generally satisfied with the information provided especially on diagnosis and diagnostic tests, treatment and overall experience but there are information needs that need to be addressed more efficiently and effectively. The findings of this study support previous research which indicates that cancer patients who are receiving treatment have many information needs. Respondents were generally satisfied with the information provided, although some discrepancies were noted which reflect the complexities associated with cancer patient education.

## Background

A diagnosis of cancer elicits psychological distress and is a very traumatic event [1–3]. The nature of the disease requires patients to learn about the illness, make difficult decisions regarding the ensuing treatment and cope with the consequences of the illness with its associated treatment side effects. Given the complex nature of the disease and the treatment modalities as well as the psychosocial impact associated with the disease, those diagnosed with cancer and their family members will encounter information and emotional support needs throughout the course of the disease and treatment. The shift to outpatient treatment has increased the necessity for a good patient education, as most patients and their caregivers now have to deal with treatment-related problems and adverse effects at home. The need for self-management of disease and treatment-related problems is compounded by the more aggressive and long-term therapies now available. These modern treatments often make patients more vulnerable to ongoing adverse effects of the treatment.

Providing cancer patients with information helps patients with decision making, prepares them for treatment and helps them cope with adverse effects associated with it, reduces anxiety and depression, increases satisfaction with treatment, improves communication with family and improves quality of life [4–11]. Patient education and meeting patients’ information needs is a fundamental aspect of patient-centred care [12]. It is only when patients are fully informed that they can become an active partner in the process of their care. As patient education is a major component of the healthcare professional’s role, patient satisfaction with information provided is one important indicator of the quality of care delivered. Patient satisfaction has been associated with improved patient adherence to medical recommendations, better outcomes, loyalty to the institution, greater willingness to recommend services to others and reduced risk of malpractice suits [13–16].

Studies have identified that cancer patients have many informational needs that vary with gender, age and type and stage of disease [4, 17–19]. These needs relate to the disease itself, its treatment and associated costs [5, 9, 17, 20–23]. Despite the number of studies on the informational needs of cancer patients, there is a little agreement on the type and amount of information patients require. In addition, many of these studies do not assess the other dimensions of information needs, such as patient satisfaction with the information received, as well as the relative importance of information needs and sources. Determining both information needs and satisfaction with its receipt can identify the gaps in information delivery, thereby assisting healthcare providers in improving support services that help cancer patients cope while undergoing treatment. Patient education that corresponds to patients’ needs, values, wishes and psychological circumstances is likely to be most effective [24], as it increases the chance that the information is correctly understood, remembered and used by patients.

The purpose of this study was to: 1) establish the information needs of cancer patients undergoing treatment; 2) identify the gap between the information they currently receive and the information they want by measuring satisfaction levels and 3) establish information sources and the patients’ preferences for them.

This paper focuses on the information needs of patients undergoing cancer treatment and their level of satisfaction with the information received. The data and further details about the study about their actual source and preferred source of information will be described and presented elsewhere.

## Methods

### Sample and setting

Patients attending the ambulatory treatment unit of the National Cancer Centre Singapore (NCCS) were invited to participate in the study. The NCCS is a tertiary medical centre that treats 70% of Singapore’s cancer patients in the public sector. Patients were considered eligible if they were: 1) diagnosed with cancer and receiving treatment; 2) able to understand and communicate in English/ Mandarin and 3) 21 years and above. Patients who were cognitively impaired, terminally ill and unaware of their diagnosis were not eligible for the study. On receiving consent to participate in the study, patients were administered the Needs assessment questionnaire in a language of their preference (either English or Chinese). Demographic, socio-economic and clinical information such as diagnosed cancer type and treatment received for it were also collected.

This study was approved by the Centralized Institutional Review Board of the Singapore Health Services and written informed consent was obtained from all respondents.

### Needs assessment questionnaire

We developed a self-reporting 76-item survey questionnaire based on an extensive review of the literature [25–31]. The items were critically reviewed by an expert panel of 15 oncology nurses. It was translated into Chinese. Following refinement of the questionnaire, we conducted a pilot study on 11 eligible patients to determine its validity. No item was deleted from the instrument during the pilot study and no further refinements were necessary.

The survey covered the following domains: 1) diagnosis; 2) tests and investigations; 3) surgery; 4) radiation therapy; 5) chemotherapy; 6) hormonal therapy; 7) clinical trials; 8) sexual aspects of care; 9) psychosocial aspect of care; 10) supportive care; 11) financial care and 12) overall experience. Respondents were asked to rate the importance of information relating to each of these domains on a 5-point Likert scale (1 = not important, 2 = slightly important, 3 = neutral, 4 = important and 5 = very important).

Similarly, satisfaction with information provided by NCCS staff on each domain of informational need was assessed using a 5-point Likert scale (1 = very dissatisfied, 2 = dissatisfied, 3 = neither satisfied nor dissatisfied, 4 = satisfied and 5 = very satisfied). The difference between perceived importance and satisfaction was considered as an indicator of gaps in information delivery.

### Statistical methods

The sample size for the study was estimated to be at least 405 patients to achieve precision level (width of 95% confidence interval) of +/-0.05 for the mean satisfaction level, assuming standard deviation (SD) = 0.5 and the data from 5% of the patients will not be useable due to incompletely filling out the questionnaire.

The importance and satisfaction level of the information needs were estimated using mean and corresponding 95% confidence intervals for each domain of the need assessment questionnaire. To account for importance level in the satisfaction level of the information, the weighted mean satisfaction level for overall experience on information received was calculated as the mean of the products of the satisfaction-and importance-level responses. The weighted mean satisfaction level for each domain of information needs was likewise calculated. Finally, all mean and weighted mean importance and satisfaction values were compared with 4 (important or satisfied) using the one-sample *t*-test. Patients who had not answered any items were excluded from that domain-specific analysis. Statistical analyses were performed using SPSS version 20.

## Results

The information on demographic is presented first, followed separately by the 12 aspects of care domains. The 12 domains are grouped and presented together based on a relatively unique and specific component of the topic or construct of interest. They are: 1) diagnosis and diagnostic test, 2) surgery, radiation therapy, chemotherapy, hormonal therapy, clinical trials, 3) sexual aspects of care, psychosocial aspects of care, supportive care and financial care and 4) overall experience.

### Response rate and characteristics of respondents

A total of 826 patients were approached from October 2015 to February 2016. 341 (41%) declined to participate, 33 (4%) patients were excluded for returning incomplete questionnaires, 12 (1%) withdrew and 29 (4%) did not return the questionnaire. This led to the final sample of 411 patients (50%). 316 responded in English (77%) and the remaining 95 (23%) responded in Chinese.

Table 1 describes the self-reported demographics, diagnosis and treatment modalities of the patient sample. The fairly educated sample consisted of Chinese, Malay and Indian and other races with Chinese making up the majority. The top three forms of cancers in Singapore (colorectal, breast and lung) are represented in the sample. Twenty-three percent (*n* = 93) had received one treatment modality and 77% (*n* = 318) received two or more treatment modalities.

**Figure 1.**
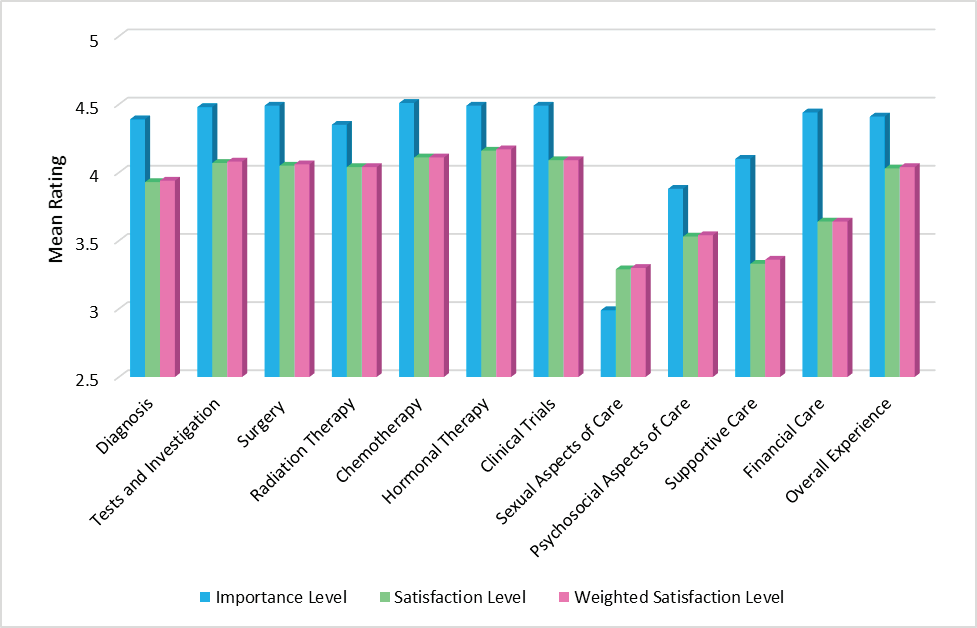
Importance and satisfaction level of respondents relating to information received overall and about various aspects of care.

Table 2 illustrates the importance and satisfaction level of respondents relating to information received about various aspects of care. A comparison of the mean weighted satisfaction scores relating to overall importance and satisfaction with regards to information needs, and for each of the 12 items relating to the various aspects of care surveyed is displayed in Figure 1.

### Diagnosis and diagnostic test

In this study, 396 and 405 respondents completely responded to items in the diagnosis and diagnostic test domains, respectively. The mean Likert scores for importance exceeded 4 (=important) for both diagnosis (mean = 4.39; *P*-value < 0.001) and diagnostic tests (mean = 4.48; *P*-value < 0.001). The mean Likert scores for satisfaction also exceeded 4 (=satisfied) for diagnostic tests (mean = 4.07; *P*-value = 0.026), but was lower than 4 for diagnosis (mean = 3.93; *P*-value = 0.035). This was caused by lower scoring for the items assessing ‘being informed about causes of the cancer of the type of cancer you have’ (mean = 3.71; *P*-value < 0.001) and ‘being informed about the survival rates for your cancer’ (mean = 3.82; *P*-value <0.001) (Table 2).

### Surgery, radiation therapy, chemotherapy, hormonal therapy, clinical trials

Responses to items asking about the importance and satisfaction levels relating to information given about cancer treatment modalities yielded favourable results, with all categories receiving a mean rating > 4 (Table 2).

### Sexual aspects of care, psychosocial aspects of care, supportive care and financial care

Table 2 shows the mean ratings for items in the importance and satisfaction domains relating to sexual, psychosocial, supportive and financial aspects of care. Mean Likert scores for the importance of sexual aspects of care indicated that respondents were generally nearly neutral in outlook (mean = 2.99; *P*-value < 0.004), while scores for psychosocial aspects of care were also lower than 4 (mean = 3.88; *P*-value = 0.021). Mean Likert scores for the importance of supportive care and financial care were also significantly higher than 4 (supportive care: mean = 4.10: *P*-value = 0.011; financial care: mean = 4.44: *P*-value < 0.001). Mean satisfaction levels for all four domains considered here were consistently lower than 4 (each *P*-value < 0.001).

### Overall experience

The mean Likert scores for the overall importance of and satisfaction with information given were 4.41 (95% CI 4.34–4.47; *P*-value < 0.001) and 4.03 (95% CI 3.97–4.09; *P*-value = 0.348), respectively, with the weighted mean satisfaction score being 4.04 (95% CI 3.98–4.10; *P*-value = 0.180). Although nine items relating to information needs in this domain had a mean Likert score greater than 4, it was lower than this threshold for the item ‘having a variety of information sources’ (mean = 3.79; *P*-value = 0.010).

## Discussion

The results of this survey support previous research which indicates that cancer patients who were currently receiving treatment have many information needs. To the best of our knowledge, the current study is among the first to examine questions specifically formulated to identify the information needs of patients who were undergoing cancer treatment by measuring the importance of the information as well as satisfaction with the information received. It is also the first study to determine the information needs of cancer patients in Singapore.

### Importance of information and satisfaction with information received as perceived by the cancer patients

Almost all patients wanted information about the disease, tests and investigations, treatment, side-effects, sexuality, psychosocial and supportive care and financial care. In addition, almost all items listed in the questions in each section were rated as important or very important.

The responses also indicate that respondents were generally satisfied with the information provided, especially those that are related to treatments. In order of satisfaction (based on mean weighted satisfaction scores), patients were most satisfied with the information received on hormonal therapy (4.17), chemotherapy (4.11) and clinical trials (4.09). However, there are aspects of information that need to be addressed more efficiently and effectively. This is reflected in the mean weighted satisfaction scores of the following aspects of care based from the lowest scores: 1) sexual aspects of care (3.3), 2) supportive care (3.36), 3) psychosocial aspect of care (3.54) and 4) financial care (3.64).

### Diagnosis and diagnostic tests

The findings indicate that respondents rated all the questions on the section on diagnosis and diagnostic tests as important and that they were satisfied with the information provided. Respondents were less satisfied with the information provided about the causes of the cancers they had and the attendant survival rates. This result agrees with those of other studies [4, 5, 9, 20, 21]. Survival outcome is a key in making decisions on treatment options [4, 5, 21] as patients need such information to respond to what are often complex decisions when outcomes can sometimes be uncertain.

Cancer patients in our study also considered information regarding tests (reason for the administration of tests, time taken for test results to be available, and when the results would be available and being an explanation of the test results) to be important and highly desirable. Such information reduces uncertainty, and therefore relieves anxiety and stress. Previous studies have similarly reported reasons for and outcomes of investigations to be desired by cancer patients [32, 33], with their lack leading to an increased level of anxiety and fear [34, 35].

### Surgery, radiation therapy, chemotherapy, hormonal therapy, clinical trials

Regardless of treatment modality, all questions on the section relating to the various types of treatment for cancer were rated as important by all respondents. These findings are supported by other studies where it was also established that the vast majority of cancer patients want a great deal of specific information concerning their illness and treatment [17] and that information needs about side effects of treatment were common [20, 21].

The present study identified some variance in scores for respondents undergoing surgery, radiation therapy, chemotherapy and hormonal therapy with regards to importance and satisfaction ratings for the item indicating ‘information with the late effects of treatment’. This item did not score as well as the other questions relating to treatment modalities. This might be due to time constraints during the consultation process leading to a greater emphasis on informing patients about the disease and treatment at the expense of issues associated with supportive care. Time and resources are critical constraints that limit the amount of information and support that can be provided in the clinical context and emphasis should be placed on how to cope with aggressive treatment regimes. These constraints highlight the importance of considering more efficient and effective methods for providing information to ensure all information needs are met.

### Sexual aspect of care, psychosocial aspect of care, supportive care and financial care

Sexuality is integral to the quality of life [36]. In this study, respondents rated information on sexuality as the least important. It was thus expected that the score for satisfaction would be higher than the importance score since patients considered sexuality the least important. The level of importance assigned to sexuality may be due to patients prioritising curing the disease and managing the side-effects of treatment over all else. Other possible reasons may be due to alteration in body image, side effects of treatment and emotional distress caused by the disease and its treatment [37, 38]. Cultural values and beliefs may also come into play here. Sexuality for some is a highly personal subject which patients may be unwilling to discuss openly with others. A study by Nie and Gao [37] involving Chinese respondents cited multiple reasons for no sexual activity including fatigue, physical problems and lack of interest. Zeng *et al* [38] established that the rate of sexual inactivity of the Chinese women surveyed was relatively high (70.5%) and the reasons for sexual inactivity were related to worrying about the possibility of weakening the potency of treatment, fear of cancer recurrence and lack of sexual interest. There may also be cultural values and beliefs that influence healthcare preferences behaviours. That is, for many people, sexuality is a personal subject that is hard to talk about openly, either with sexual partners or with healthcare professionals. However, it is interesting to note that the mean weighted satisfaction score is the lowest score as compared to the other aspects of care, demonstrating the complexities and challenges in dealing with this topic. It is therefore important to assess patients to find out whether they have any concerns with any aspect of sexual care, identify barriers to communication about such issues and deliver the information in accordance to patients’ needs.

Although respondents rated information about ‘supportive care’ as important, the overall satisfaction was the lowest score after ‘sexuality’. Questions on ‘being informed about the types of food/supplement that are good for you’; ‘being informed about things you can do to help yourself get well’; ‘being informed about cancer survivor support services’ and ‘Being informed about the multi-disciplinary care clinics where your entire team discusses your case’ were rated as important but the satisfaction level was rated lower than that for importance.

Respondents also wanted more information about types of food and supplements that are good for them and complementary therapies. Cancer and its treatment can affect the nutritional intake and the general health of the patients. Food has important social and cultural meaning, and there are many foods that are considered taboo when a patient is diagnosed with cancer, especially in Asian cultures [39, 40]. At the same time, patients also showed a preference for taking dietary supplements, whose intake during cancer treatment is controversial and can be potentially dangerous as they may counteract the effect of drugs. These supplements include traditional Chinese medicine, vitamin and mineral supplements and antioxidants [41]. Therefore, information about the types of food/supplements including traditional Chinese medicine that may be beneficial would help patients consider appropriate food and supplements to boost their general well-being and prevent nutritional deficits. In addition, information about food/supplements that are detrimental due to the possibility of drug interactions must also be discussed as patients with cancer had a high tendency to use herb or vitamin/minerals [42]. A study in 2012 on the use of complementary and alternative medicine (CAM) in adult patients with cancer at the same institution revealed that 66.7% (*n* = 146) reported using CAM and 80.8% used natural products which include herbal products, special diets, dietary supplement and vitamins and minerals. The study also revealed that only half of the participants informed their medical doctor regarding CAM use, demonstrating poor communication between patients and their healthcare providers, on the use of CAM.

Though respondents rated satisfaction level with the information received on ‘potential side effects caused by cancer treatment and how to cope with them’ highly, the question on ‘being informed about things you can do to help yourself get well’ was rated low in terms of satisfaction level. This finding could indicate that patients with cancer prefer to be independent and not be a burden to their caregivers and one way to be more self-reliant is to learn about self-care measures/activities to get well. This may include information about types of physical activities that would help to improve their physical and functional wellbeing.

This study also indicated that respondents wanted more information about cancer survivor support services. Accurate information and support from counsellors and other survivors can empower the patient, relieve anxiety and improve quality of life and this information gap is frequently present. In a study by Ernstmann *et al* [43], almost a third of the cancer population surveyed indicated an unmet need for psychosocial support. Concerns about social functioning, role functioning and emotional functioning in descending order were present in both male and female patients. Respondents were also anxious and depressed. Psychosocial support was needed for emotional functioning.

This study supports the view that an unmet need exists and more attention should be given by healthcare professionals to recognising anxiety and depression early on so that appropriate counselling and support can be given. In addition, there is a need to look into more publicity about education programs and support group meetings.

Due to the complex nature of cancer and its treatment, a multidisciplinary team approach is important to provide comprehensive care. Although the study setting provides multidisciplinary care as standard practice, this study revealed that more awareness of such services is needed.

The majority of respondents rated information about financial information relating to costs of treatment and financial support as important. A study by Veenstra *et al* [22] established that the financial burden is high, particularly among those who use adjuvant chemotherapy. Sherwood *et al* [23] also established that participants expressed a great deal of worry regarding financial matters and the impact of treatment costs on their families. Participants were concerned with issues such as the future impact of current economic choices, their ability to make ends meet and being able to afford current cancer care. As the majority of patients (77%) of respondents in this study received two or more treatment modalities, economic hardships can be a major concern and these can arise from high costs for treatment, lack of insurance coverage and loss of income. It is therefore important to address their information needs relating to the financial costs of cancer treatment and sources where financial support is available may help alleviate their worry, and stress.

### Overall experience

Respondents in this study felt that all aspects of the information delivery under this section were important and they were satisfied with how the information was being delivered. The question on ‘having a variety of information sources (e.g., leaflet and audio materials)’ scored the lowest score (3.79) as compared to all the other questions.

It is not surprising that respondents wanted access to a variety of information sources. Information retention is an issue due to the emotional and physical effects of the disease and its treatment. There is evidence that patients retain only 20% of what they hear [44]. Being an ambulatory centre, access to help with regards to problems encountered after the Centre’s operating hours may be restricted. Written patient information materials are found to be a useful tool in contributing to patient knowledge [45, 46]. Therefore, written information materials/videos are useful supplements as they can help reinforce oral information/teaching provided by the healthcare professionals. When used effectively, they can help health care professionals maximise limited teaching time and enable patients to better manage their health and improve their coping skills and well-being.

### Limitations

There are limitations to the study. First, the convenience sample was recruited from only one ambulatory cancer site in Singapore and may not represent the population at large. Another limitation is the high rejection rate; therefore, there could be a nonresponse bias. It is possible that the perspective of this group was not fully explored and we may have under-estimated the degrees of unmet information needs. Nevertheless, the centre is the largest cancer centre in Singapore and sees about 70% of all public cancer patients. Moreover, we obtained responses from a reasonable cross-section of adult cancer patients with tumour types that are the top cancers in Singapore.

This study also precluded those cancer patients who were not aware of their diagnosis. The nondisclosure of information of the diagnosis of cancer is still practiced in the local population where protective truthfulness is requested by some family members.

In addition, though the study consisted of both English and Mandarin speaking participants and may have excluded the Malay and Indian races, there were 16.1% Malays and Indians who participated in this survey. This is generally representative of the population as Chinese made up 74.3% of the resident population in 2015, while the Malays constituted 13.3% and the Indians formed 9.1% [47].

The investigators’ study instrument was developed based on the extensive literature review instead of a validated questionnaire. All items listed in the 12 components measuring the various aspects of care indicated that these items are important to the cancer patients, suggesting some face validity. The questionnaire was also verified by a panel of 15 oncology nurse experts and a pilot study on both the Chinese and English versions of the questionnaire was conducted. These data did not yield any discrepancies or need to include additional information in the survey, suggesting that common relevant questions have not been overlooked.

## Conclusion

A diagnosis of cancer is a catastrophic event and affects all aspects of life for patients. Cancer treatment has also become more complex and aggressive. It is, therefore, crucial to identify and address the information needs of cancer patients in order to help them make decisions and cope, potentially improving their satisfaction with the services received and health outcomes. Determining what information is important to cancer patients and how satisfied they are with the information they receive is critical in helping healthcare professionals identify areas in which improvement is needed and targeting education programs to meet these needs. This would enable better resource allocation and help healthcare institutions in delivering patient-centred quality care.

The results of the study indicate that cancer patients undergoing cancer treatment have many information needs. They particularly want information about their disease, treatment, investigation results, psychosocial and financial aspects of care. The findings also suggest that respondents were more satisfied with information on diagnosis and diagnostic tests, treatment, and how information was delivered but generally less satisfied with the information received concerning sexual, psychosocial, supportive and financial aspects of care. Due to the time constraints during the consultation process, compromise may be made in relation to information delivery. Healthcare professionals may place more emphasis on informing patients about the ‘more critical information’ such as disease and treatment. Such constraints highlight the importance of considering more efficient and effective methods for providing information to ensure all information needs are met. This includes the creation of patient-centred resources such as educational materials, support services and communication tools specific to cancer patients.

The study also identified some discrepancies in ratings of importance and satisfaction levels for items such as ‘sexual aspects of care’. As this study was not designed to evaluate specific reasons for the importance of any single informational domain, further studies would help to establish the reasons for these discrepancies.

To the best of our knowledge, the present study is among the first to examine unmet information needs not only in terms of patient satisfaction with the information received but also in terms of the importance of the information for patients. It is also the first study to determine the information needs of cancer patients in Singapore.

## Practice implications

The results of this study have highlighted the type of information cancer patients undergoing treatment need in order to help them with decision making and coping skills. This can serve as guidelines for information delivery as well as creating patient-centred support and resources.

## Conflicts of interest

The authors have no financial conflicts of interest to declare.

## Funding

This research was made possible by a grant from the Community Cancer Fund [COMCF-YR2015-MAY-NS1].

## Figures and Tables

**Table 1.** Characteristics of survey respondents.

Characteristics, *n* (%)		*N* = 411
Sex		
	Male	153 (37.8)
	Female	259 (64.0)
Age (years)		
	21–40	45 (10.9)
	41– 60	223 (54.3)
	60 and above	142 (34.5)
	Unknown	1
Ethnicity		
	Chinese	317 (77.1)
	Malay	46 (11.2)
	Indian	20 (4.9)
	Others	27 (6.6)
	Unknown	1
Marital status		
	Married	320 (77.9)
	Non Married	85 (20.7)
	Unknown	6
Highest level of education		
	Primary or less	81 (19.7)
	Secondary/higher secondary	213 (51.8)
	Tertiary	109 (26.5)
	Unknown	8 (2)
Type of treatment (*n* = 411)		
	Surgery	248 (60.3)
	Radiation therapy	118 (28.7)
	Chemotherapy	404 (98.3)
	Hormonal therapy	45 (10.9)
	Clinical trials	62 (15.1)
Employment status		
	Employed	176 (42.8)
	Unemployed	230 (56.0)
	Unknown	5
Cancer type		
	Breast	115 (28.0)
	Colon/rectal	60 (14.6)
	Lung	52 (12.6)
	Others	171 (41.6)
	Unknown	39
Disease status		
	Newly diagnosed	304 (74.0)
	Recurrent	98 (23.8)
	Unknown	9
Treatment modality		
	One	93 (23.0)
	Two or more	318 (78.5)

**Table 2.** Importance and satisfaction level of respondents relating to information received about various aspects of care.

Aspects of care Items of care	Importance level	Satisfaction level	Satisfaction level weighted by importance level of item of care
	Mean (95% CI)	Mean (95% CI)	Mean (95% CI)
Diagnosis (*n* = 396)	4.39 (4.31, 4.46)	3.93 (3.86, 3.99)	3.94 (3.88, 5.01)
Being informed about which type of cancer you have	4.50 (4.42, 4.58)	4.11 (4.03, 4.19)	
Being informed about the causes of the type of cancer you have	4.30 (4.21, 3.39)	3.71 (3.62, 3.79)	
Being informed about the variety of treatment options available in terms of managing your cancer	4.48 (4.40, 4.56)	4.08 (4.01, 4.15)	
Being informed about the survival rates for your cancer	4.27 (4.17, 4.37)	3.82 (3.73, 3.90)	
Tests and investigations (*n* = 405)	4.48 (4.41, 4.55)	4.07 (4.01, 4.14)	4.08 (4.01, 4.14)
Being informed about the purpose of the tests (e.g., computed tomography scans, magnetic resonance imaging scans and blood tests)	4.46 (4.39, 4.54)	4.11 (4.04, 4.18)	
Being informed about when you can expect the results	4.42 (4.34, 4.49)	4.03 (4.95, 4.10)	
Being explained about the results of the tests	4.56 (4.50, 4.63)	4.09 (4.01, 4.16)	
Surgery (*n* = 238)	4.49 (4.40, 4.58)	4.05 (3.96, 4.14)	4.06 (3.97, 4.14)
Being informed about the possible benefits or harms of your cancer surgery (including its success rate)	4.53 (4.44, 4.62)	4.11 (4.01, 4.21)	
Being informed before the operation for how you would feel afterwards	4.50 (4.41, 4.60)	4.07 (3.97, 4.17)	
Being informed about the time period of recovery and how you can be an active participant in your treatment	4.48 (4.39, 4.58)	3.99 (3.88, 4.10)	
Being informed about the possible late effects of surgery	4.44 (4.33, 4.55)	3.92 (3.81, 4.03)	
Being informed on how to care for yourself physically post-surgery	4.45 (4.36, 4.55)	4.07 (3.97, 4.17)	
Being informed about what you should or should not do after you leave the hospital/centre	4.48 (4.39, 4.57)	4.08 (3.98, 4.17)	
Being informed about who to contact if you are worried about your condition or treatment after you leave the hospital/centre	4.54 (4.45, 4.63)	4.12 (4.02, 4.21)	
Radiation therapy (*n* = 118)	4.35 (4.20, 4.50)	4.04 (3.92, 4.15)	4.04 (3.93, 4.16)
Being informed about the possible benefits and harm of radiation therapy (including its success rate)	4.33 (4.16, 4.50)	4.07 (3.93, 4.20)	
Being equipped with enough information for decision-making about whether you should receive radiation therapy	4.34 (4.18, 4.50)	4.02 (3.89, 4.14)	
Being informed about the potential side effects caused by radiation therapy and how to cope with them	4.40 (4.24, 4.55)	4.04 (3.92, 4.17)	
Being informed about the duration of the entire radiation therapy and how you can be an active participant in your treatment	4.36 (4.21, 4.51)	4.15 (4.04, 4.27)	
Being informed about the possible late effects of radiation therapy	4.34 (4.17, 4.50)	3.93 (3.79, 4.07)	
Being informed about who to contact regarding any problems you may have after you leave the centre	4.35 (4.19, 4.51)	4.00 (3.86, 4.14)	
Chemotherapy (*n* = 402)	4.51 (4.44, 4.58)	4.11 (4.04, 4.17)	4.11 (4.05, 4.18)
Being informed about the possible benefits and harm of chemotherapy (including its success rate)	4.52 (4.44, 4.59)	4.10 (4.03, 4.17)	
Being equipped with enough information for decision-making about whether you should receive chemotherapy	4.51 (4.43, 4.58)	4.10 (4.02, 4.17)	
Being informed about the potential side effects caused by chemotherapy and how to cope with them	4.57 (4.50, 4.63)	4.18 (4.11, 4.25)	
Being informed about the duration of the entire chemotherapy treatment and how you can be an active participant in your treatment	4.50 (4.43, 4.57)	4.14 (4.07, 4.21)	
Being informed about the possible late effects of chemotherapy	4.45 (4.37, 4.53)	3.95 (3.87, 4.03)	
Being informed about who to contact regarding any problems you may have after you leave the centre	4.53 (4.45, 4.60)	4.17 (4.10, 4.24)	
Hormonal therapy (*n* = 44)	4.49 (4.29, 4.69)	4.16 (3.95, 4.37)	4.17 (3.96, 4.37)
Being informed about the possible benefits and harm of hormonal (including its success rate)	4.46 (4.20, 4.71)	4.09 (3.81, 4.38)	
Being equipped with enough information for decision-making about whether you should receive hormonal therapy	4.50 (4.28, 4.72)	4.16 (3.93, 4.39)	
Being informed about the potential side effects caused by hormonal therapy and how to cope with them	4.50 (4.30, 4.70)	4.21 (3.97, 4.44)	
Being informed about the duration of the entire hormonal therapy treatment and how you can be an active participant in your treatment	4.48 (4.28, 4.68)	4.18 (3.96, 4.40)	
Being informed about the possible late effects of hormonal therapy	4.46 (4.25, 4.66)	3.96 (3.65, 4.25)	
Being informed about who to contact regarding any problems you may have after you leave the centre	4.55 (4.35, 4.74)	4.34 (4.14, 4.54)	
Clinical Trials (*n* = 60)	4.49 (4.30, 4.69)	4.09 (3.93, 4.24)	4.09 (3.93, 4.24)
Being informed about the eligibility for clinical trials	4.42 (4.20, 4.63)	4.07 (3.88, 4.26)	
Being informed about the potential risks and benefits of this treatment	4.53 (4.33, 4.73)	4.15 (3.99, 4.31)	
Being informed about your rights as trial participants	4.48 (4.28, 4.69)	4.10 (3.93, 4.27)	
Being informed about the differences between standard treatment and this treatment	4.50 (4.30, 4.70)	3.98 (3.79, 4.18)	
Being informed about the process and procedures of clinical trial	4.47 (4.26, 4.67)	4.05 (3.88, 4.21)	
Being informed about the duration of the entire trial and how you can be an active participant in your treatment	4.50 (4.30, 4.70)	3.98 (3.80, 4.17)	
Being informed about whether you could quit halfway after you participated	4.50 (4.30, 4.70)	4.20 (4.05, 4.35)	
Being informed about who to contact regarding any problems you may have after you leave the centre	4.55 (4.35, 4.75)	4.15 (3.98, 4.32)	
Sexual aspect of care (*n* = 341)	2.99 (2.84, 3.15)	3.29 (3.20, 3.37)	3.30 (3.21, 3.38)
Being informed about the effects of cancer treatment on your sexuality or fertility	3.20 (3.04, 3.36)	3.37 (3.28, 3.46)	
Being informed about whether having sex during treatment can be harmful to you or your partner	3.08 (2.92, 3.24)	3.27 (3.18, 3.36)	
Being informed about when you could or could not have sex	3.03 (2.87, 3.19)	3.26 (3.17, 3.35)	
Being informed about the options that can help in your fertility (e.g., sperms banking and embryo storage)	2.85 (2.68, 3.01)	3.28 (3.19, 3.36)	
Being informed when you can start a family or have children after the cancer treatment	2.80 (2.63, 2.97)	3.26 (3.17, 3.34)	
Psychosocial aspect of care (*n* = 391)	3.88 (3.78, 3.98)	3.53 (3.45, 3.62)	3.54 (3.46, 3.63)
Being informed about the psychological and social problems caused by the types of cancer you have and its treatment	3.84 (3.72, 3.95)	3.44 (3.35, 3.53)	
Being informed about the services at NCCS for dealing with psychological and social problems caused by cancer you have and its treatment	3.89 (3.77, 4.00)	3.47 (3.37, 3.56)	
Being informed that the cancer is not contagious	3.92 (3.80, 4.03)	3.70 (3.60, 3.79)	
Supportive care (*n* = 410)	4.10 (4.02, 4.18)	3.33 (3.25, 3.41)	3.36 (3.28, 3.44)
Being informed about the types of food/supplement that are good for you	4.46 (4.38, 4.53)	3.53 (3.43, 3.63)	
Being informed about things you can do to help yourself get well	4.42 (4.34, 4.49)	3.56 (3.46, 3.66)	
Being informed about complementary therapies (e.g., traditional Chinese medicine and art therapy)	3.98 (3.88, 4.09)	3.21 (3.11, 3.30)	
Being informed about supporting resources available (e.g., support group and counsellors)	3.94 (3.83, 4.05)	3.33 (3.23, 3.43)	
Being informed about the patient education services and classes	3.78 (3.66, 3.89)	3.17 (3.08, 3.27)	
Being informed about cancer survivor support services (e.g., counselling, screening and follow-up care for survivors)	4.07 (3.97, 4.16)	3.28 (3.18, 3.38)	
Being informed about the multi-disciplinary care clinics where your entire care team discusses your case	4.06 (3.96, 4.15)	3.22 (3.12, 3.32)	
Financial care (*n* = 396)	4.44 (4.37, 4.52)	3.64 (3.55, 3.72)	3.64 (3.56, 3.73)
Being informed about the costs of your treatment(s)	4.49 (4.40, 4.57)	3.72 (3.63, 3.81)	
Being informed about the coverage by benefits/extended medical insurance	4.42 (4.34, 4.50)	3.59 (3.49, 3.68)	
Being informed about Medisave, Medishield and Medifund and how they apply to your situation	4.46 (4.38, 4.54)	3.68 (3.59, 3.78)	
Being informed on how to access financial support or advice	4.40 (4.32, 4.49)	3.55 (3.45, 3.65)	
Overall experience (*n* = 411)	4.41 (4.34, 4.47)	4.03 (3.97, 4.09)	4.04 (3.98, 4.10)
Receiving information from NCCS professionals, throughout your experience as a cancer patient	4.43 (4.36, 4.50)	3.97 (3.89, 4.04)	
Having the opportunity for your family or yourself to talk to a doctor	4.51 (4.44, 4.57)	4.14 (4.07, 4.21)	
Receiving a sufficient amount of information to help you make a decision or cope with the disease and treatment	4.48 (4.41, 4.55)	3.99 (3.91, 4.07)	
Receiving information that was easy to understand	4.49 (4.43, 4.55)	4.07 (4.00, 4.14)	
Receiving information at the time when you need it	4.45 (4.38, 4.52)	3.97 (3.90, 4.05)	
Receiving information in the language you understand	4.48 (4.42, 4.55)	4.17 (4.10, 4.23)	
Receiving information that was sufficiently sensitive to and respectful of your culture?	4.08 (3.97, 4.18)	3.99 (3.92, 4.05)	
Having questions answered honestly	4.50 (4.44, 4.56)	4.11 (4.05, 4.18)	
Having enough time to ask the doctor/nurse or healthcare professionals questions	4.51 (4.45, 4.57)	4.09 (4.02, 4.16)	
Having a variety of information sources (e.g., leaflet and audio materials)	4.13 (4.03, 4.23)	3.79 (3.71, 3.87)	

CI: Confidence interval; NCCS: National Cancer Centre Singapore

Bold values represent the mean values less than 4.0 with *P*-value < 0.05 for comparison with 4.0 using the one-sample *t*-test.
